# Congenital Deafness and Deaf-Mutism: A Historical Perspective

**DOI:** 10.3390/children11010051

**Published:** 2023-12-30

**Authors:** Andrea Cozza, Valerio Maria Di Pasquale Fiasca, Alessandro Martini

**Affiliations:** 1 Department of Cardiac, Thoracic, Vascular Sciences and Public Health, University of Padua, 35128 Padua, Italy; 2 Section of Otorhinolaryngology—Head and Neck Surgery, Department of Neurosciences, University of Padua, 35128 Padua, Italy; valeriomaria.dipasqualefiasca@studenti.unipd.it; 3 Padova University Research Center “International Auditory Processing Project in Venice (I-APPROVE)”, Department of Neurosciences, University of Padua, 35128 Padua, Italy; alessandromartini@unipd.it

**Keywords:** congenital deafness, deafmutism, otology history, audiology history

## Abstract

Hearing loss is the most common sensory deficit and one of the most common congenital abnormalities. The estimated prevalence of moderate and severe hearing loss in a normal newborn is 0.1–0.3%, while the prevalence is 2–4% in newborns admitted to the newborn intensive care unit. Therefore, early detection and prompt treatment are of utmost importance in preventing the unwanted sequel of hearing loss on normal language development. The problem of congenital deafness is today addressed on the one hand with hearing screening at birth, on the other with the early (at around 3 months of age) application of hearing aids or, in case of lack of benefit, by the cochlear implant. Molecular genetics, antibody tests for some viruses, and diagnostic imaging have largely contributed to an effective etiological classification. A correct diagnosis and timely fitting of hearing aids or cochlear implants is useful for deaf children. The association between congenital deafness and “mutism”, with all the consequences on/the consideration that deaf mutes have had since ancient times, not only from a social point of view but also from a legislative point of view, continued until the end of the nineteenth century, with the development on one side of new methods for the rehabilitation of language and on the other of sign language. But we need to get to the last decades of the last century to have, on the one hand, the diffusion of “universal newborn hearing screening”, the discovery of the genetic causes of over half of congenital deafness, and on the other hand the cochlear implants that have allowed thousands of children born deaf the development of normal speech. Below, we will analyze the evolution of the problem between deafness and deaf-mutism over the centuries, with particular attention to the nineteenth century.

## 1. Introduction

«[…] *Men who are deaf from birth are also always mute: they can emit vocal sounds, but not articulate a language*» (*Historia animalium*, IV 9, 536 b 3–5) [1]. Aristotle (384–322 BC) was already clear on the relationship between congenital deafness and language development: in the absence of a normal hearing function, there is no normal development of language.

The association between congenital deafness and “mutism”, with all the consequences on the consideration that deaf mutes have had since ancient times, not only from a social point of view but also from a legislative one, continued until the end of the nineteenth century, with the development on one side of new methods for the rehabilitation of language and on the other of sign language. Albert Eulenburg (1840–1917), in one of the most widespread Encyclopedic Dictionaries of the late nineteenth century, writes, «*Deafness is congenital or acquired. Congenital deafness always results in deaf-mutism*» [2].

But we need to get to the last decades of the last century to have, on the one hand, the diffusion of “universal newborn hearing screening”, the discovery of the genetic causes of over half of congenital deafness, and on the other hand, the cochlear implants that have allowed thousands of children born deaf the development of normal speech [3,4,5,6,7].

## 2. Antiquity and Middle Ages

The so-called “medical” papyri of ancient Egypt, among the various aspects treated, describe elements of anatomy and otological pathology [8,9]. The ear is *mesedher,* and annotations, especially of a pathological nature attributable to it, are mainly found in the Ebers Papyrus (1550 BC), in the Edwin Smith Papyrus (1550 BC, but with glosses that refer to a much older text dating back to 2300 BC) and in the Berlin Papyrus (1300 BC). The Berlin Papyrus refers to otological problems in children, giving therapeutic indications in case of probable otitis media ear pain [8,9], but in general, in the sources of ancient Egyptian medical literature, there seem to be no clear and explicit references to congenital deafness. In the Ebers Papyrus, however, a correlation between deafness and muteness seems to be highlighted, even if there is no additional information: «*When he is deaf, then he cannot speak*» [9]. Furthermore, the Akkadians left medical tablets in which there is a probable reference to congenital deafness: «*If it rests on the lower abdomen, it will give birth to a deaf person*» [10].

In classical antiquity, sight and hearing significantly affected the intellectual speculations of philosophers as the cardinal senses of life [11]. The knowledge of the ear and hearing is broadened thanks to the observations of Hippocrates of Cos (460–377 BC) both in anatomical and pathological terms [12]. Hippocrates, however, in his works inserts references to deafness, or rather to hearing loss, but mostly to that of an acquired and transitory type because of infectious diseases or, in any case, in the presence of acute fever [10]. Hippocrates also realized that the deaf from birth cannot speak.

Furthermore, in the Greek world, the works of the philosopher Plato (427–347 BC) are permeated with references, especially in a metaphorical sense, to sight and hearing and their lack or deficiency, even if sight holds a privileged position compared to hearing [13]. Furthermore, Plato, in his dialogue *Cratylus*, clearly testifies to the ability of mute people to communicate through non-verbal language. The protagonists of this work by Plato are Cratylus, Hermogenes, and Socrates. The latter, speaking with Hermogenes, states: «*Answer this: if we had neither voice nor tongue and we wanted to make things clear to each other, we would not try, as the mute do now, to manifest them with the hands, the head and all the rest of the body?*» [14].

Hearing and deafness are subsequently investigated by the philosophical-scientific gaze of Aristotle of Stagira, whose works are imbued with reasoning and analyses of the sense of hearing [15]. In fact, according to Aristotle, hearing is intimately correlated with memory and learning, and this sense seems to have a privileged position compared to sight precisely in favoring learning itself [16,17,18]. The Stagyrite, therefore, notes how congenital deafness and mutism are interconnected and that the development of verbal language is subordinated to the ability to hear. This is interesting if we consider how, in classical antiquity, greater emphasis was always given to the inability to speak with an articulate voice rather than the ability to hear [10,18,19].

Numerous textual sources and various archaeological artifacts testify to the diffusion of otological pathology in the Roman world. In various archaeological contexts, anatomical ear-shaped votive offerings have been found, presumably created, and used to thank the divinity for the healing of certain otological ailments [20]. Through the reading of tomb inscriptions, we know that there was a specialist doctor for ear care: the *medicus auricularius.* Galen, in his *De locis affectis*, identifies the cause of deafness in the lesion of the “nerve proper to hearing” [21]. In Galen’s works, however, specific references to congenital deafness do not seem to exist, if not a terminological connection present in the work *In Hippocratis de officina medici commentarii III* [19].

During the long period of the medieval era, ideas and concepts of medicine of the classical era were revived, in which the pathology is mainly traced back to the imbalance of the four cardinal humors or to traumas and external causes [22]. According to Rhazes (865–925 AD), hearing deficits result from defects of the hearing nerve [22]. The writer and translator John Trevisa (1342–1402), in his work *Dialogus inter dominum et clericum* (1387), stated that those who are deaf (from birth) are always also mute and that learning to speak occurs through hearing: whoever is deaf is always also mute since they cannot hear words and learn to speak [23]. During the ancient age, the deaf mute individual was affected by a very heavy social stigma [10], just as during the Middle Ages, he was in a condition of social isolation [10,23].

## 3. Modern Age

In the modern era, there is a greater sensitivity and attention towards people with deafness, and it began to take into consideration the possibility and methods of expression by deaf mute people. There are also testimonies of cases in which people with deaf-mutism, who for centuries could not enjoy civil rights, were admitted to participate in notarial deeds [24]. Pedro Ponce de León (1520–1584), a Spanish Benedictine monk, was among the very first who imparted education to deaf mutes, above all through writing and the use of a manual alphabet [10,25,26]. Again, in Spain, Juan Martin Pablo Bonet (1579–1633) and Manuel Ramírez de Carríon (1579–1652) also distinguished themselves in teaching the deaf mutes. The effectiveness of the teachings that allowed deaf mutes to communicate was mentioned by the physician Ezechiel de Castro (also known as Pedro) in his work *Il colostro*, published in Verona in the first half of the seventeenth century. De Castro, in fact, mentioned some cases of “illustrious” deaf mutes («*there are numerous examples in Spain, of mute children either by nature, or by accident of a notable cascade*») who, above all thanks to de Carríon’s teachings, were able to communicate: «*they speak vocally, and clearly while remaining deaf, but not mute*» [27].

In England, one of the first to advocate hand sign language was John Bulwer (1606–1656). Specific treatises on the education of English deaf mutes also began to be published [25]. Other Anglo-Saxon contributions are those of John Wallis (1616–1703) [10,25]. In the second half of the seventeenth century, however, the correlation between deafness and dumbness was always underlined, especially if the former is congenital: «*Whoever is inclined from birth to deafness, gradually even falls to dumbness*» [28]. Still, the Swiss physician Johann Konrad Amman (1669–1724), in teaching deaf mutes, gave greater importance to spoken language than to manual sign language. He taught his students to articulate sounds with the vibrations of the larynx perceived with the fingers as a guide [25].

## 4. The XVIII and XIX Centuries

At the end of the modern age, Jacob Rodriguez Pereira (1715–1780), Charles Michel de l’Epèe (1712–1789), Roch-Ambroise Cucurron Sicard (1742–1822), George Raphael (1673–1740), Johann Ludwig Ferdinand Arnoldi (1737–1783) and Samuel Heinicke (1727–1790) dealt with deaf-mutism and education of deaf mutes in various capacities. In Italy in the eighteenth century, among the first, Tommaso Silvestri (1744–1789) was one of the first to teach deaf mutes; they trained at Charles de l’Epée, learning the methodology, and founded a school for deaf mutes in Rome [10,25,26].

Even if the distinction between congenital and acquired deafness occurred in previous centuries, it was in the first half of the nineteenth century that, with the birth of a new medical-surgical discipline centered on ear diseases, Otology [25,29], which began to be clarified the etiological causes of profound deafness.

Certainly, a great impact on the diffusion of otological knowledge was played by Sir William Robert Wills Wilde’s book *Practical Observations on Aural Surgery and the Nature and Treatment of Diseases of the Ear [30], published in 1853 both in Dublin and* Philadelphia, and in Germany in 1855.

Wilde (1815–1876) addresses the problem of congenital deafness and deaf dumbness from the point of view of incidence in the population, and this “epidemiological” approach will take an important role throughout the century.

Wilde writes: «*My official position as one of the Irish Census Commissioners has not only afforded me peculiar means for investigating this subject, but has also directed my attention to it in an especial manner; and I have reason to believe that minute inquiry into all the circumstances relating to mutism which has been lately carried on in this portion of the United Kingdom, is not only the most correct which has yet been undertaken in any country but is such as to throw much light upon the statistics, and the social, moral, and physical condition of that class of our fellow—creatures deprived by congenital malformation, accident, or disease, of the faculty of hearing, and, in consequence thereof, of the powers of speech*» [30].

According to Wilde: «*They it must, however, be remembered, suffer simply from their privation; the deaf, in addition, often labor under the most harassing noises, and from partially hearing what is said, without being able to understand the purport of general conversation, and being, moreover, much confused by the Babel of sounds around them, should claim more sympathy than is generally awarded them*» [30].

## 5. Epidemiology and Aetiology of Congenital Deafness in the Nineteenth Century

The population of deaf mutes in Germany was surveyed in 1871 by Georg von Mayr, the foremost representative of German administrative and bureaucratic statistics. At the time, 152,751 were affected out of a total population of 206,304,081 (prevalence: 0.07%) [31]. This figure was also stable in the subsequent population census of 1875, although it is not entirely clear whether the surveys adopted distinctions between patients judged dumb for other etiology and real deaf mutes. The data described a much higher frequency in Europe (7.81 cases per 10,000 inhabitants) than in the United States of America (4.20 cases per 10,000 inhabitants), with notable differences between the various European regions [2].

More authors of the time, and even later in the twentieth century [12,32], observed how the frequency of deaf-mutism increased in alluvial and mountainous areas [33]. They hypothesized the socio-economic condition of the great poverty of the Alpine population as an etiological factor («*Poverty and deprivation, bad food and unclean, poorly ventilated homes*») [34,35]. According to von Mayr, another risk factor, considering the greater frequency in the Danube basin and in northern Germany, was alluvial soil. A further observation concerned the increase in the incidence of acquired deafness in the regions affected by an epidemic of cerebrospinal meningitis, which raged in 1864–1865 in different areas of Germany [36,37], in 1870 in Norway [38] and in 1882 in Italy [39]. August Lucae [40] and Arnold Ludwig Gotthilf Heller [41] recently described infectious affections and inflammation of the internal ear. The pathogenesis was attributed to ischemic damage of the labyrinth. The theories of Voltolini proposed, on the contrary, an inflammatory damage, with consequent loss of function of the auditory organ [42]. Among the causes of congenital deaf-mutism, authors such as Arthur Freiherr von Tröltsch [43] and others proposed defective embryonic development [44,45]. Unfortunately, data regarding the frequency of these infections are often incomplete or unreliable.

Among other causes were enlisted parental alcohol abuse or mental illness, high age difference between parents, and psychological disturbs of the pregnancy. Various authors described cases of association of deafness with the absence of internal ear structures [46]. There is also already known a possible association between these anomalies and the anomalies of the external and middle ear, like atresia, fibrosis, or adherences. Moreover, Salomon Moos described cases of congenital deaf-mutism in children affected by intrauterine infections [45,47].

The study of histological alteration of internal ear spread at the beginning of the XX century. First studies in post-mortem specimens were available and showed anomalies in the internal ear cavities, which were occupied by bone partially or completely. Similar histological alterations were described in both congenital and acquired deaf mutes [48], showing a difference in localization and intensity. These affections were usually divided into “congenital malformations” and “regressive alteration”, which were difficult to distinguish due to the limited knowledge and available tests. A study published by Klaus Wittmaack [49] demonstrated the same affections described in acquired deaf mutes in experimental labyrinthitis. Manasse proposed to consider “congenital” the alterations of the internal ear, which showed a change in the cochlear skeleton, which depended on anomalies of the cochlear nerve [47]. Gorke noticed that the spiral lamina was described as highly in acquired forms of deaf-mutism, so they proposed its alteration as a criterion for diagnosing congenital deaf-mutism [50]. Alexander defined the condition as “hereditary-degenerative deaf-mutism” [51].

## 6. Possible Role of Consanguinity and Inheritance

Consanguinity was proposed as a risk factor by observing the higher rate of deaf-mutism in the areas with the highest frequency of marriages between blood relatives, such as rural or mountain areas [34,35,52,53]. This higher frequency was also documented in some closed social groups with a tendency towards union among the same members.

A particular aspect of these “epidemiological” investigations concerns religious confession. Richard Liebreich and von Mayr report in Germany a higher incidence of deaf-mutism among Jews compared to Christians (ratio between 1:2 and 1:4) and, albeit with a smaller difference, a higher incidence among Protestants compared to Catholics [31,54]. These differences were justified by the high number of consanguineous marriages among Jews; consanguineous marriages were permitted among Protestants and prohibited among Catholics. Schmaltz observes how the frequency of deaf-mutism increases with the number of consanguineous spouses [35].

However, Eulenburg clearly raises the problem of consanguinity both among the inhabitants of the mountainous areas («*close proximity of life and due to the often-deficient exchange with the outside world*») but also among the Israelites, in whom a great frequency of deaf mutes had been found. The question is raised here whether consanguinity is the risk factor or the fact that «*among those who enter into marriage no other individual dispositions yet exist; inheritance* etc. *for the procreation of deaf and dumb children*» [2].

An important piece of information that was considered sufficient to suspect a congenital form was the presence of other conditions in familiars, such as other cases of deaf-mutism or certain ocular illnesses (retinitis pigmentosa). Congenital syndromes or malformations, such as retinitis, syndactyly, epilepsy, chorea, and cleft lip, were known to be associated with deafness [2]. An indirect familiar connection was described by different authors, with no clear direct inheritance [35,36]. It was described as a more frequent familiarity in the collateral line than vertical and has been hypothesized to be a recessive transmission.

Various authors such as Knapp, Moos, Menière, and Liebreich examined the incidence of deaf mutes in consanguineous marriages. [44,52,55]. According to Eulenberg, «*the question of the inheritance of congenital deaf-mutism is not truly resolved with all certainty through statistics*» and takes into consideration the possibilities of direct and indirect hereditary transmission [2]. Holger Peter Theodor Mygind [56], Wilhelm Uchermann [38], and Arthur Hartmann [36] are among the first to strongly consider the hereditary cause; according to these authors, the consanguinity of the parents does nothing but strengthen the inheritance when it exists.

At the beginning of the 20th century, the existence of risk factors that influenced the frequency of deaf-mutism was already a subject of debate. No clear causes of congenital deaf-mutism were discovered. Giuseppe Gradenigo, in 1903, writes, «heredity has a great influence in the production of congenital deaf-mutism,» and the existence of other cases of deaf-mutism in the family or of pigmentary retinitis are criteria to support the congenital form [57].

The data on the frequency before the age of two are not clear due to the technological limits of the diagnostic systems, the reluctance of families to accept this diagnosis, and the limits of the census systems of the time. More reliable estimates belong from the 5th year, schooling age [2]. Considering the methods of that time, it was not possible to make an early diagnosis, and the little patients usually received a deaf-mutism diagnosis at two years of age or later or did not receive a diagnosis at all. Tests available for deaf-mutism diagnosis depended on the collaboration of the little patient, causing a problem of reliability among such tests: direction of the head towards the source of the sound or change of expression. The study of the thresholds at different frequencies was carried out through choristers, whistles, or bells [12].

The discussion regarding the role of consanguinity continued until the second half of the twentieth century. In this regard, the discussion reported in the proceedings of a 1969 symposium is particularly interesting to understand the knowledge of the causes of deafness and the role of heredity and consanguinity in the 1970s. Fraser reported on a large sample of 3534 individuals «*who have been profoundly deaf from childhood*,» describing many syndromic cases and paying particular attention to the causes based “*on family history*” [58]_._ In the discussion, Ronald Hinchcliffe (Professor of Audiological Medicine at Royal National Throat Nose & Ear Hospital in London) asks Fraser: «*Dr Fraser, did I take you to imply that genetic counseling to discourage near relatives who are deaf from marrying would not appreciably reduce the prevalence of genetically determined hearing loss?*». The question is not that far from Graham Bell’s position on eugenics a century earlier. Fraser’s response is very interesting: «*The first is that discouraging deaf people from marrying each other will reduce the prevalence of deafness very little where the deafness is genetically determined and is recessive. Such deafness is not normally inherited from one generation to the next because it is caused by so many different genes…More often, transmission of deafness from parent to child is because one of the parents has dominant deafness, and this parent may transmit deafness to his or her children whether the marriage partner is hearing or deaf».* Fraser concludes: «*I feel that when our ignorance of aetiological mechanisms is remedied, treatment may have as great role as counseling in prevention.*» Fraser was referring to Edward Allen Fay’s enormously important study *Marriage of the Deaf in America,* published in 1898, in which he found that the proportion of deaf children was not greater if both parents were deaf or only one was deaf [59].

## 7. Treatments of Congenital Deafness

Although in 1853 Wilde had stated that «*Nervous deafness must be treated according to its cause*» [30], at the end of the nineteenth century and the first decades of the twentieth century, in medical treatises, including specialized ones, complete deafness of deaf mutes was questioned (Snickers, director of the Institute for the Deaf mutes of Liège and Magnat of the Péreire Institute in Paris, stated that deaf mutes often speak correctly in their sleep) and it was still confused with the “hysterical” forms.

The first treatment proposed for deaf-mutism was the prophylactic strategy. It consisted of limiting the possible causes, such as improving social conditions, avoiding marriages between relatives, treating infectious diseases such as tuberculosis and syphilis, reduction of alcoholism. Other treatments were pointed towards the causes of acquired deaf-mutism, such as otitis media and nasopharynx illnesses (accounting for 20% of children treated in deaf mutes institutes) [60].

Once the deafness and the subsequent mutism were established, no treatment was able to solve the condition. Scientists such as Urbantschitsch proposed a treatment aiming to improve the use of hearing residuals.

For those affected by high levels of hearing loss, a possible rehabilitation was using some instruments to amplify sound, even though they achieved poor results. Among those are artificial eardrums such as the Yearsley eardrum (1848), the Toynbee eardrum (1854), or ear trumpets [61].

At the end of XVIII, electrical treatment was proposed for deaf people [62]. Alessandro Volta was one of the first who tried electrical stimulation of the inner ear using two metal probes on himself [63]. He described this procedure in a letter to Luigi Valentino Brugnatelli in 1802 [64]. In this letter, he also mentioned the use of an electro-motor apparatus in Jever, Germany, where Johann Justus Anton Sprenger developed a method to electrically stimulate hearing in deaf people [65]. The electric flow was applied to the tragus, mastoid, and auditory external channels on both ears. Volta described the successful use of this method on a young deaf woman who became capable of hearing sounds after the treatment. Other scientists investigated the use of electrical treatment for hearing restoration [66,67,68]. Vincenzo Cozzolino (1853–1911) in 1886 performed an experiment in which it was possible to transfer the ability to speak from a speaking woman to a mute one using electromagnetic stimulation [61].

## 8. Education of Affected Children

It is difficult to collect certain data on the education of the deafmutes. The first mention of this issue dates to the XV century: Rudolph Agricola (1443–1485), in his De Inventione Dialectica, referred to a case of a deafmute who was able to learn to write and read [69]. Later the first who defined a systematic education for this kind of patient was Pedro Ponce de Leon. He left us no information regarding his methods, but his contemporaries reported that he was able to teach us how to write and read. Pablo Bonet was the first to write a text regarding the education of the deafmutes (*Reduction de las letras y arte para ensenar a ablar a los mudos*—in English: *Reduction of letters and arts to teach the mutes how to speak*) in 1620 [70]. Other scientists, such as John Bulwer (1606–1656), John Wallis (1616–1703), William Holder (1616–1698) and George Dalgarno (1616–1687) taught phonetic and symbolic language. Conrad Amman (1669–1724), a Swiss doctor, concentrated his work on the articulative method: his method was based on showing the deafmutes the position of the mouth; he also let the patients comprehend the sounds produced by the doctor’s larynx by touch (as lately applied in the phonetic system). In France, the abbot De L’Epée (1712-1789) was known for his efforts in deafmutes instruction. He and his successor, Sicard, were fervent supporters of the symbolic language. Moreover, he was the founder of the first French institute for deafmutes education in Paris. The French method was also applied in Austria, where the first instruction institute was founded in 1779 by W. Stork.

Particular attention to the “educational” aspect of the deaf and dumb was developed in German-speaking countries in the central years of the nineteenth century. In 1856, Meissner’s treatise *Taubstummheit und Taubstummenbildung* (*Deaf-Mutism and Education of the Deaf-mute*) [71] was published. Hartmann’s monumental treatise was then published in 1880 with the same title, *Taubstummheit und Taubstummenbildung* (*Deaf-Mutism and Education of the Deaf mute*) [36]. Holger Mygind’s 1895 treatise had a particular influence and was immediately translated into many languages [72].

The results of these institutes spread fast, so many other structures were founded throughout the whole of Europe and beyond. In the late XIX century, specific structures for education were active (364 in North America, Europe, Brazil, and Japan), even though they were not enough to provide proper education to the entire deaf mute population. Most European countries could not ensure education for most of their deaf mute population (Russia, Portugal, Italy, Spain, Austria). On the other hand, countries such as Germany, Belgium, and the Netherlands had better results. The instruction of the deafmutes started at seven years old and lasted 8 years. The educative methods were mostly the phonetic and the symbolic language.

Two kinds of education were applied for deafmutes at the end of the XIX century: French education and German education. In the first one, sign language was used as the first language; in the second one, applied by Simon Heincke (1727–1790), the first founder of a deafmutes instruction institution in Lipsia, Germany, the phonetic language was taught to the deafmutes, along with the lip-reading. Another type of language is taught in Spain, France, and the United Kingdom: the digital language, or manual, if practiced with two hands. It consisted of teaching the deaf mutes how to sign each letter with their hands. The deafmutes also learned how to read and write. In this period, the scientific community agreed on the use of phonetic language, which was considered the only way to provide the deafmutes with a complete culture. This was stated in the “International congress of the deafmutes teachers”, hold in Milan in 1880: «*Ogni sordomuto che non sia affetto da idiotismo e sia generalmente capace di una coltura, deve essere istruito -per mezzo del metodo articolativo, presupposto che il tempo della istruzione venga corrispondentemente prolungato ed il piano della istruzione venga adattato alle attitudini del discente*» (In English: «*Every deaf mute who is not affected by idiocy and is generally capable of learning must be taught by means of the articulative method, provided that the time of instruction is correspondingly prolonged and the plan of instruction is adapted to the aptitudes of the learner*») [73].

Around the year 1880, deaf mutes were reported to be occupied in different jobs at a rate. They most frequently worked in factories, mines, and agriculture [2].

## 9. Conclusions

The discussion on the approach to the problem of congenital deafness and the consequent lack of language development has continued for millennia. Only the development, which has occurred in the last thirty years of diagnostic methods applied from birth and, above all, the new rehabilitation possibilities through early hearing aids and possibly cochlear implantology, have allowed thousands of children born with severe/profound deafness to develop normal oral language. The development of clinical genetics, molecular genetics, and epigenetics has certainly contributed to improving knowledge of deafness and possible innovative treatments [74,75,76,77].

But it is clear that even when interest in the problem of deaf-mutism increased in the nineteenth century, deaf people lived on the margins of society, and the treatment reserved for them was often the result of an attitude made up of misunderstandings and sometimes cynicism on the part of the medical class. The awareness of the development of knowledge and, above all, the different positions that have developed over time is also important for respect due to people with a pathology that has such an impact on everyday life.

## Data Availability

Not applicable.
